# Advocates of climate action? The age of members of parliament and their activity in legislative debates on climate change

**DOI:** 10.1007/s44168-022-00017-2

**Published:** 2022-07-12

**Authors:** Marc Debus, Noam Himmelrath

**Affiliations:** School of Social Sciences and Mannheim Centre for European Social Research, University of Mannheim, A5, 6, 68131 Mannheim, Germany

**Keywords:** Politics of climate change, Parliamentary debates, German Bundestag, Age of Members of Parliament, Representation, Climate-change policy

## Abstract

Parliamentary debates are an important stage in the process of designing new policies and play an important role for discussing the policy reactions to exogenous events like the COVID-19 pandemic or long-term developments like climate change. We combine theories on vote-seeking strategies of political actors with theoretical accounts that highlight the impact of personal characteristics of politicians and argue that in particular younger Members of Parliament (MPs) should put more emphasis on issues related to climate change and its consequences than older MPs. We test our hypotheses on the basis of an original dataset covering all parliamentary debates that focus on issues related to climate change in the German Bundestag from 2013 until 2021, thus concentrating on a time period when climate change became a highly salient issue among the German public. We find that the age of MPs matters: the younger MPs are, the more speeches they contribute to parliamentary debates related to climate policy.

**Graphical Abstract:**

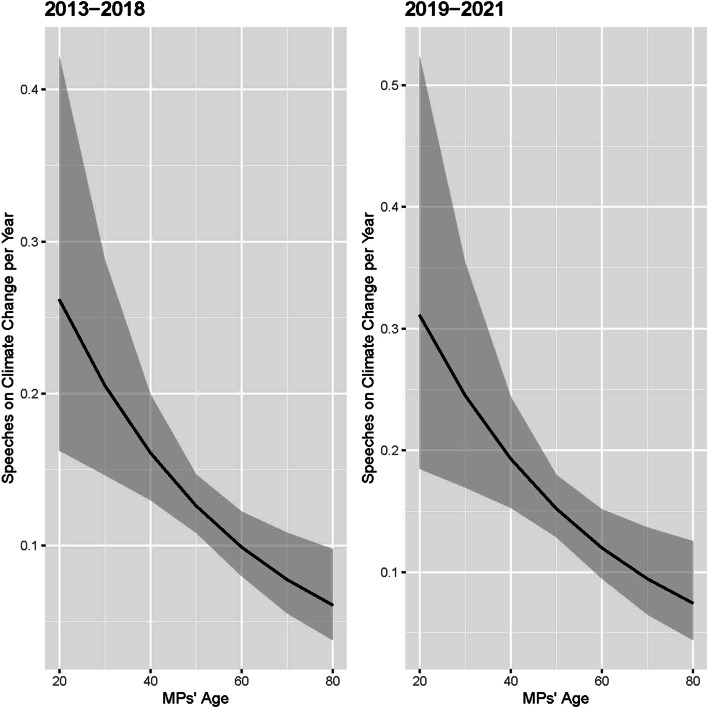

## Introduction

Parliamentary debates are an important stage in the process of convincing political decision-makers as well as for designing new policies and play an important role in discussing the policy responses to highly dangerous processes like climate change. Moreover, debates in parliaments and legislatures influence the public and decision-making processes among citizens. For instance, one unusual side-effect of Brexit and related discussions around it was that BBC parliament, a TV channel which mostly broadcasts the debates in the British Houses of Parliament, reached more viewers in a week in January than MTV in the UK (see Bäck et al. 2021a: 1). Political representatives were able to present their positions on Brexit in these debates, propose solutions to the stalemate on British and European politics, and communicate their policy proposals to the other MPs and the public.

Given this prominent role of parliaments and their members in the policy cycle, in particular during the stages of agenda-setting, policy formulation, and policy adoption (e.g., Andeweg and Nijzink 1995; Bräuninger and Debus 2009; Bräuninger et al. 2017; Knill and Tosun 2020), the composition of parliaments both in terms of the partisan affiliation of MPs but also their personal characteristics is an important aspect when it comes to the content of new policies. Therefore, adequate parliamentary representation is one of the corner stones of modern liberal democracy which promises its citizens a pluralistic opinion formation process by elected political actors. However, many societal groups are descriptively underrepresented in parliaments worldwide. Especially in contexts where these groups are affected by exogenous phenomena and substantive legislative outcomes, questions about the promised adequate representation arise. Short-term exogenous shocks, such as the COVID-19 pandemic, but also long-term developments, such as the global climate crisis, present different challenges to different generations. While vaccination campaigns against the COVID-19 virus initially focused on elderly and adults, the youth was left aside, but heavily affected by the COVID-19 pandemic since nurseries, kindergartens, schools, and universities were often closed or practiced online teaching. As older people represent a significantly higher share of the electorate and are more likely to vote (e.g., Goerres 2008), such decisions by elected politicians in parliaments and governments are not surprising: vote-seeking parties and their representatives should be more likely to take the preferences of citizens into account which are eligible to vote and are actually more likely to cast a ballot when drafting policy proposals and acting and deciding in parliament and government.

Recognizing that personal characteristics of elected representatives like MPs matter for legislative behavior in specific contexts that address moral or ethical issues (e.g., Baumann 2018; Burden 2007; Euchner and Preidel 2017; Searing 1994), we argue in this contribution that younger elected representatives should focus on topics that are more important to younger people. In particular, the issue of climate change as a long-term exogenous shock will have drastic implications on future living. Among the multiple threats, the rapid acceleration of climate-related disasters, for instance, will disproportionately affect the lives of today’s young people compared to older generations. Decisions made today will have lasting impact on future generations. Furthermore, concentrating on an issue which a significant share of citizens considers a highly important problem, a promising strategy for promoting the individual career, in particular for younger MPs, can be preparing and presenting policy proposals in parliament, for instance by giving speeches. Therefore, younger MP could — simply for career-seeking incentives — become advocates of climate action.

Descriptive representation in parliaments should be linked to substantive representation, that is, the mere presence of representatives who are characteristically similar to their constituents changes policy outcomes into the preferred direction of their supporters (Phillips 1995). In that vein, the parliamentary under-representation of women (Wängnerud 2009), ethnic minorities (Bird et al. 2010), and the working class (Carnes 2012) have been widely studied as examples of inadequate descriptive representation of certain societal groups that affects policy outcomes (e.g., Homola 2021; Kittilson 2011; Koch and Fulton 2011). One group that arguably presents a special case and has so far been mostly overlooked is the youth. Young people are descriptively underrepresented in parliaments worldwide (Stockemer and Sundström 2018). To shed light on the descriptive parliamentary representation of young people, we here focus on how age influences the parliamentary behavior and actions of MPs on a highly important and pressing issue like climate change. We conceptualize age as a sociodemographic variable that, in line with research on such characteristics, should affect legislative behavior, independent of other important conditions. To that end, we ask if the age of MPs affects their participation in parliamentary debates in this policy area that should be highly salient for younger people.

We proceed by, first, formulating a theoretical argument, before providing a descriptive overview of the patterns of representation of young people in the German parliament (Bundestag). We focus on Germany in the empirical section since environmental protection in general and climate change in particular became a highly salient issue in Germany in the last decade. By using data on parliamentary debates from the Bundestag since 2013, we subsequently demonstrate that not only all MPs tend to give more speeches on climate-related issues since this topic — as we will show — became increasingly important among the German population, while comparative studies show that climate policy salience varies substantially between countries and is positively related to country wealth (Crawley et al. 2021). Moreover, we find evidence for our claim that in particular younger MPs participate more in parliamentary debates on climate change, even when controlling for a variety of further key explanatory variables. We conclude that personal characteristics of MPs matter for legislative behavior and thus for climate policy, in particular when the context of a parliamentary debate allows MPs to gain profile within their party and among the public. Younger MPs can thus indeed be seen as advocates of climate action who bring this issue onto the political and parliamentary agenda — also induced through career-seeking incentives.

## Literature review and theoretical argument

Sociodemographic characteristics of legislators have an effect on the preferences of MPs and consequently on their parliamentary actions. In that regard, scholars have investigated the effects of MPs’ gender (Catalano 2009; Höhmann 2020; Reynolds 2013) and MPs’ migration background (Saalfeld and Bischof 2013), as well as candidates’ disabilities (Reher 2022). Many of these studies consider MPs’ age as an influential variable and incorporate it in their estimations. Surprisingly, however, the age of MPs as a separate independent variable has so far been mostly overlooked in academic research. Only few researchers have addressed the underrepresentation of the youth in the political decision-making process. Most recently, Sundström and Stockemer (2021) have introduced a new concept to measure youths’ underrepresentation in parliaments. The authors find that young adults under the age of 35 are generally underrepresented by a factor of three, lending support to the fundamental claim that young people are descriptively underrepresented in parliaments. In the case of Germany, which we focus on in this paper, the MPs of the 18th and 19th Bundestag, who were elected in 2013 and 2017, had an average age of 52.83 and 50 years, respectively, at the time of the election. Overall, there were only small differences between the parties in the complete dataset, with the liberal Free Democratic Party (FDP) having the youngest parliamentary group (overall average age: 47.19 years) and the Social Democrats (SPD) the oldest group (overall average age: 52.16 years). By contrast, the average age of the German population in 2017 was only 44.4 years.

This discrepancy is the main focus for many academic approaches: most of the existing research focuses on how many young MPs are present in legislative chambers, how to increase this number (Stockemer and Sundström 2018), and how selected theoretical concepts affect the share of young MPs in parliaments (Stockemer and Sundström 2019). We here take a different approach: Instead of asking how a more adequate descriptive representation of the youth can be guaranteed, we investigate how the existing underrepresentation translates into legislative behavior. Research has shown a mismatch between voters’ preferences for younger politicians and the overrepresentation of older politicians in parliaments and governments (Eshima and Smith 2021). We argue that this represents a strategic opportunity for young MPs to behave differently in parliamentary processes. Their personal characteristics are thus likely to result in a specific legislative behavior and, more concretely, in an interest for specific policy domains. Previous studies provide evidence for the effect of MPs’ age on their legislative behavior in the parliament of the Czech Republic, indicating that age and tenure indeed can significantly influence MPs’ legislative behavior and processes of decision-making in parliaments (Hájek 2019). In the same vein, we argue that younger MPs make strategic use of their age to signal their sincere interest on a highly salient issue like climate change because of career-seeking incentives.

We derive our expectation on the basis of two theoretical perspectives. In line with existing theoretical accounts (e.g., Müller and Saalfeld 1997; Strøm 1997, 2012), we argue, first, that a parliamentarian’s behavior is likely to be determined by the level of competition for reselection, renomination, and reelection for posts within the party and/or legislative offices. As the attainment of the latter goals is contingent on successful renomination and reelection, the goals can be ordered hierarchically. In fact, “[the] iron-clad necessity of election in democratic legislatures […] makes the ‘single-minded pursuit of reelection’ the primary instrumental goal of legislators” (Strøm 2012, 90). Accordingly, MPs’ decisions should be mainly determined by the desire to maximize the likelihood of reelection. Secondly, we combine this perspective with the literature on personal characteristics of MPs and theories that focus on the strategic positioning of parties and their representatives on salient issues (see also Baumann et al. 2015).

On the basis of the office seeking motivation, the legislative behavior of parties on the one hand and their individual representatives on the other is usually explained as being strategic, whereby differences in these strategies are largely assumed to be rooted in the institutional level of politics like the electoral system (e.g., Bol et al. 2021; Ohmura et al. 2018; Zittel and Nyhuis 2021). However, contextual features such as issues that dominate the public agenda also matter for decision-making processes of parties and individual politicians (e.g., Hobolt and De Vries 2015; Meyer and Wagner 2016; Rovny and Whitefield 2019). We therefore expect that MPs represent the preferences that prevail among their constituents through the MPs’ legislative behavior. Such a behavior inside and outside the parliament should increase the chances that MPs receive a higher level of public attention and support.

However, not every issue or topic is likely to fulfill the goal of public visibility. An important aspect is that the respective politician should be perceived by the electorate as trustworthy and/or competent to deal with and tackle problems related to that topic. One simple strategy to be perceived as competent is to link the respective issue to the personal characteristics of an MP. Needless to say, MPs are not only influenced through external factors. Recent research analyzing the decisions of individual MPs has theoretically argued and empirically shown that the legislative behavior of MPs is shaped not only by pressure from their constituents and party, but also by their own personal background like gender, family status, religious denomination, or professional background (e.g., Baumann 2018; Burden 2000, 2007; Searing 1994). For instance, MPs with a migrant background are more active in legislative debates if the topic of the debate focuses on issues related to the interests of migrants (Bäck and Debus 2020; Saalfeld and Bischof 2013). Likewise, female MPs give more speeches in policy domains that reflect stereotype women’s interests (e.g., Bäck and Debus 2019; Blumenau 2021; Hargrave and Blumenau 2021), either because of the strategic interests of the respective MPs or because their party forces those MPs with a particular personal background to be more active in related policy areas for vote-seeking reasons.

When a topic such as climate change receives a high level of issue attention in the public, an office-seeking MP — that is, an MP who seeks renomination and reelection — should try to gain a publicly visible profile on that very issue if his or her personal characteristics make him or her a trustworthy and sincere advocate of that particular issue. In addition, the party of the respective MP is also likely to benefit also from such a strategy of an individual politician, as the party as a whole should be more likely to be seen as competent in a salient topic if the respective party has representatives that are perceived as experts in a policy domain considered to be highly important by the voters. While in the case of climate change several personal characteristics like an MPs’ professional background could be helpful to link the respective MP with the climate change topic, we here focus on the MPs’ age as a very simple and straightforward personal characteristic of parliamentary representatives. Younger MPs can more easily and more sincerely argue that they (and their — planned — family) are personally affected by climate change and will therefore push policies that will tackle climate change and will reduce its negative effects. We therefore expect that — in a time period where climate change has become and continuous to be a highly salient issue among the public, in particular among younger citizens as the ‘Fridays for Future’ movement shows (Parth et al. 2020; Wallis and Loy 2021; see also Berker and Pollex 2021) — younger MPs should give more speeches in the parliament on debates related to climate change, regardless of other important variables like party affiliation or membership in parliamentary committees. Owing to these career-seeking incentives, younger MPs should be more likely to become advocates of climate action and should prioritize this issue in their parliamentary activities.

**Fig. 1:**
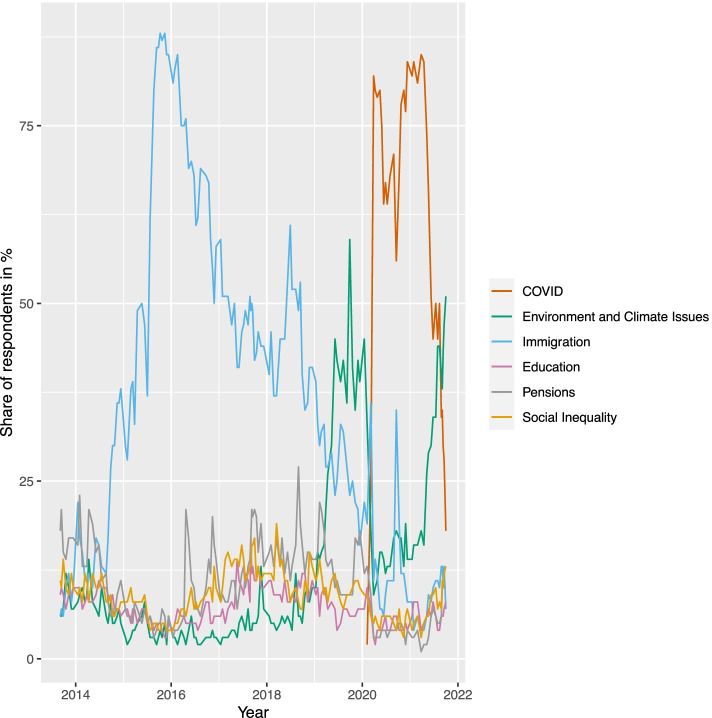
Share of respondents that consider the listed issues as the most important ones in Germany. Source: Aggregated survey data of the Forschungsgruppe Wahlen (https://www.forschungsgruppe.de/Umfragen/Politbarometer/Langzeitentwicklung_-_Themen_im_Ueberblick/Politik_II/)

To test this expectation, we make use of an original dataset that covers information on the number of speeches that members of the German Bundestag contributed to parliamentary debates related to the climate change issue in the time period between 2013 and 2021. We thus cover two legislative periods of the Bundestag with the legislative period between 2017 and 2021 characterized by a significant increase in public attention for climate issues (see Fig. 1). While environmental issues in general and climate issues in particular were mentioned by less than 10% of Germans as the most important problem until 2019, this issue was considered to be the most pressing one in 2019 before the outbreak of the COVID-19 pandemic. Since then, it did not disappear from the public agenda; instead, it became — despite the COVID-19 pandemic — again the most important problem by the end of 2021.

The wide time span during which the issue saliency for climate change increased in the public sphere additionally allows to examine whether all actors represented in parliament increase their attention to climate change questions. The latter would be expected by theoretical (and empirical) accounts that highlight the role of the public agenda for responsive changes in the programmatic profile of parties and their representatives (e.g., Adams and Somer-Topcu 2009; Hobolt and De Vries 2015; Meyer and Wagner 2016; Rovny and Whitefield 2019). However, a general strategy of parties to focus more on climate change because of an increase in issue attention among the public is not at odds with our basic argument that younger MPs should deliver more speeches on climate issues. Because parties would benefit from younger MPs who can make a sincere argument that they care about climate policy, we expect that MPs from all parties give more speeches in debates on climate change, but give way — or even encourage — younger members of their parliamentary groups to deliver speeches on climate policy. The next section provides an overview on the data and the methodological strategies used to answer our research question.

## Data and methods

We rely on speeches delivered in the German Bundestag during its 18th and 19th legislative period, covering a time span from October 22, 2013, until September 26, 2021. Our empirical model includes data from 1312 observations, where one observation equals an MP per legislative period, and a total number of 57,818 speeches. These speeches include only contributions with a minimal length of 25 words, thus excluding short interventions, disruptions, questions, and procedural introductions.^1^ Of all remaining speeches, we classify 1140, roughly 5.7%, as speeches on climate change and related issues. To identify speeches on the topic of investigation, we apply a dictionary-based approach that automatically classifies contributions during a legislative debate as speeches on climate change if at least ten occurrences of the predefined keywords are mentioned in the individual speech^2^ (Grimmer and Stewart 2013). We expect that 12 word stems are most frequently used in the context of debates on climate change.^3^ The dictionary was developed in a multi-step process. A very basic and initial list of words was adopted on the basis of existing environment-related dictionaries (Laver and Garry 2000) and further developed by manually adding relevant terms inspired by the coding scheme of the *Comparative Agendas Project* (Bevan 2019). Therefore, the debates we identify as related to climate change are also to some degree related to environmental issues. In a subsequent step, computational text analysis in the form of locally trained word embeddings was applied to identify previously missed keywords. Word embeddings use vectors to express the semantic meaning of words, based on the fact that similar words are typically numerically close and semantically related (e.g., neighboring words) and thus spatially proximate.

**Fig. 2:**
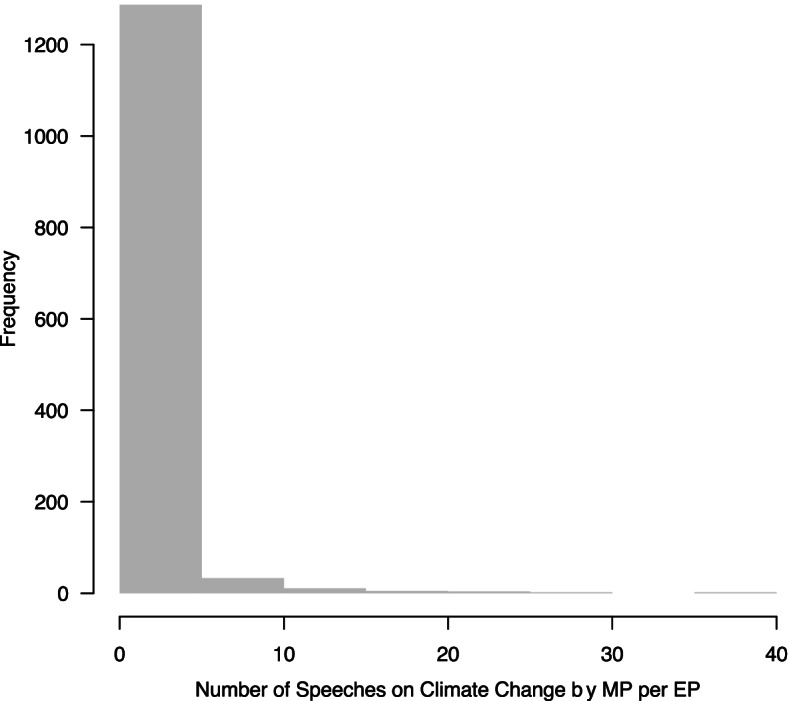
Distribution of the dependent variable

After identifying all speeches on the issue of climate change, we aggregate the data on an MP-level. Note that many MPs have been reelected in 2017. For these legislators, we differentiate between the two legislative periods to account for changing structural covariates. That is, one observation is one MP per legislative period. The dependent variable provides information on the number of speeches MPs delivered in debates on climate change in one legislative period. Figure 2 provides an overview of how many speeches the individual MPs gave and shows that a vast majority of MPs gave only some speeches on climate issues if any, whereas only very few MPs speak very often — up to 36 times — in parliamentary debates on climate change.

**Fig. 3:**
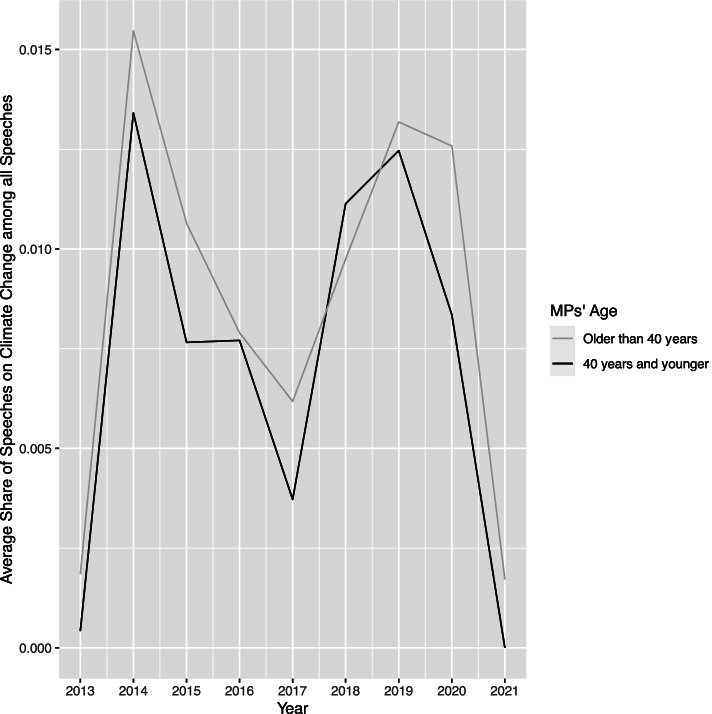
Share of speeches related to climate change in the Bundestag over time, differentiated by age group of MPs

An adequate method for analyzing a dependent count variable with a right-skewed distribution (as shown in Fig. 2) is a negative binomial regression model, used, for instance, by Proksch and Slapin (2012) in their analysis of parliamentary debates. Our main independent variable is the MPs’ age. As age is constantly changing, there are different ways to measure the variable. Here, we consider age to be dynamic and as such model MPs’ age as their age in years on the day they gave a speech. As we aggregate the dependent variable, we also do so for the MPs’ age. Hence, the main independent variable for each observation is the MP’s average age on the day s/he gave a speech in a debate related to climate change. Figure 3 below shows the average share of speeches on climate change by differentiating between MPs that are older than 40 years or that are up to 40 years old. As expected, the number of speeches among both age groups increased after 2017 when climate change became a highly salient topic among German citizens. However, there is — according to these descriptive data — no evidence that younger MPs speak more than older MPs. Moreover, we can observe a relatively high share of debates on climate issues in 2014. This could be related to debates in the German parliament on global agreements aiming at fighting climate change, but also due to the fact that 2014 was the hottest summer in Germany since the beginning of weather recordings (see https://www.dwd.de/EN/climate_environment/climatechange/_functions/news/150102_hottest_year_2014.html), thus making climate issues tangible for citizens and their representatives.

While this descriptive analysis demonstrates that younger and older MPs tend to follow the issue attention of the public, multivariate methods are required to evaluate if there is a significant difference between younger and older MPs when it comes to the number of speeches they give in debates on climate change. Since multiple variables exist that could have a confounding impact on the variable under investigation, it is crucial to control for these. First, we control for the age structure in the MPs’ electoral districts by incorporating information into the empirical models on the share of citizens under the age of 35 years (due to data availability). The ‘younger’ the constituents of an MP are on average, the more likely s/he should focus on a topic like climate change since it is of high relevance in particular for younger citizens. This data was made available by the German Federal Election Office (www.bundeswahlleiter.de) and stems from 2012 and 2015, respectively. We also control for a variety of institutional factors and further individual characteristics of MPs. For instance, the models include information on the Bundestag committee membership of the MP and provides information if an MP was a member of the committee on environment and climate protection. Furthermore, the model covers information whether the MP was elected directly in a district by a plurality of votes or through a party list. Additionally, the model controls for the MP’s gender and for the respective MPs’ party seat share in the Bundestag. In model 2, the latter variable is replaced with information on the partisan affiliation of MPs, allowing for the evaluation if a ‘green agenda’ on climate issues is in particular introduced by the representatives of the German Green Party. We also include a dummy variable that takes the value of 1 when the MP was part of the Bundestag in the previous legislative period and 0 otherwise. Lastly, we control for the economic situation in the MPs’ election district by including in the regression models the unemployment rate in the specific districts.

## Results

Table 1 presents the results of three negative binomial regression models. The dependent variable is the number of speeches an individual MP gave in debates on the issue of climate change in a legislative period. In contrast to the first model, model 2 replaces the seat share of the MP’s parliamentary parties with the party affiliation of the MPs to analyze whether MPs from parties that emphasize a ‘green agenda’ (Carter et al. 2018; Debus and Tosun 2021) speak significantly more often than MPs belonging to other parliamentary party groups. Model 3, by contrast, includes a variable that provides information on the role of MPs inside the parliamentary party group, that is, if they are in a leadership role or ordinary MPs.

**Table 1:** Determinants of the number of MPs’ speeches in debates on climate change

	Model 1	Model 2	Model 3
Age	− 0.021^**^ (0.009)	− 0.015^*^ (0.009)	− 0.020^**^ (0.008)
Pop. share below 35 years in electoral district	0.020 (0.025)	0.005 (0.026)	0.020 (0.025)
Committee member	2.412^***^ (0.238)	2.455^***^ (0.236)	2.458^***^ (0.234)
AfD		0.252 (0.451)	
FDP		0.918^**^ (0.494)	
Greens		1.175^***^ (0.342)	
SPD		0.198 (0.237)	
The Left		0.272 (0.350)	
MP directly elected	0.303 (0.220)	0.392 (0.241)	0.321 (0.217)
Female	0.205 (0.178)	0.201 (0.183)	0.231 (0.176)
Reelected	0.870^***^ (0.187)	0.813^***^ (0.202)	0.800^***^ (0.185)
Parl. group leader			1.605^**^ (0.645)
Party seat share	− 0.021^***^ (0.007)		− 0.018^**^ (0.007)
Unemployment rate in electoral district	− 0.038 (0.031)	− 0.044 (0.032)	− 0.029 (0.031)
Constant	− 0.537 (1.117)	− 0.885 (1.141)	− 0.805 (1.107)
Observations	1,312	1,312	1,312
Log likelihood	− 1146.473	− 1142.633	− 1141.829
Theta	0.160^***^ (0.015)	0.164^***^ (0.015)	0.166^***^ (0.016)
Akaike Inf. Crit.	2310.029	2311.266	2303.659

Standard errors in parentheses. Significance levels: *p < 0.1, **p < 0.05, ***p < 0.01. Members of the parliamentary party group of the Christian Democrats form the reference group in model 2

We find a robust negative effect of MPs’ age on the number of parliamentary speeches by MPs held in debates related to climate change. In all three models, the effect of the age variable is significantly negative. In substantive terms, this means that younger Bundestag MPs indeed talk more often on the climate change issue, as we hypothesized in the theoretical section of this contribution. There are mixed effects from several contextual variables: the age structure in the MPs’ electoral district has no effect on how often directly elected MPs speak in debates on climate change. Furthermore, the degree of economic problem pressure in the electoral district that MPs represent in parliament also has no effect either, nor does the difference between directly and listwise elected MPs. As expected, we find that MPs who are members of the related committee speak more in debates on climate change, as do only MPs of the Green Party, which makes sense from the perspective of the literature of issue ownership (e.g., Spoon et al. 2014; Tavits and Potter 2015). That the finding also applies to the FDP — a party emphasizing market-liberal economic policies — seems surprising; yet, the FDP emphasizes positive effects of the free market for technical innovations helping to fight climate change. The fact that the age of MPs matters for their focus on climate issues, even when controlling for an important variable like party affiliation signals support for our main argument that younger MPs from all parties are using their age strategically to be associated with an increasingly important issue like climate change.

**Fig. 4:**
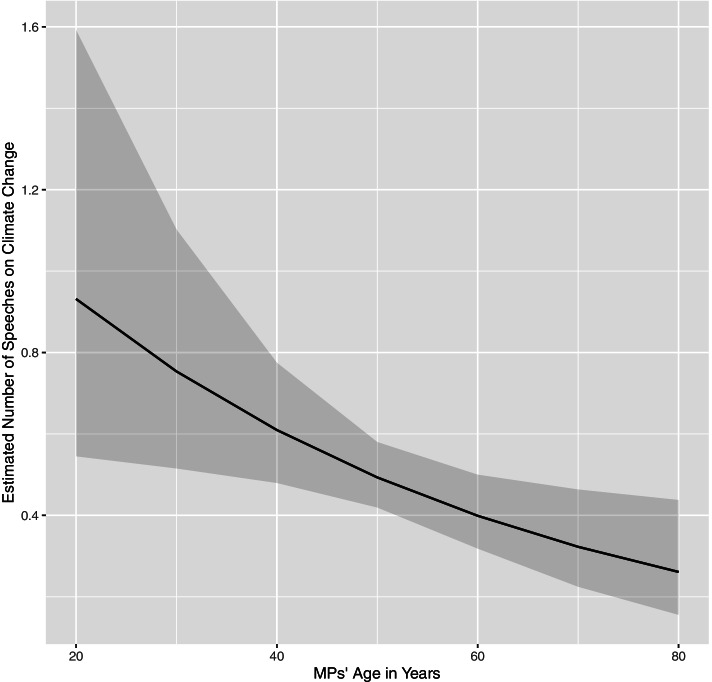
Predicted number of speeches in parliamentary debates on climate change, depending on the age of MPs (the grey shaded area shows the 90% confidence interval)

Figure 4 shows the substantive effect of MPs’ age on their participation in debates on climate change in the Bundestag from 2013 until 2021. Younger MPs are predicted to give about one speech on climate change during a legislative period, while older MPs are predicted to give only 0.3 speeches. Needless to say, an MP cannot give a third of a speech, indicating that most older MPs do not give any speech on the topic of climate change at all. This predicted number of speeches in debates on climate change in the Bundestag demonstrates the statistically significant and substantive effect of the age variable. It appears that indeed younger MPs make use of their age to gain a sincere profile on the climate change issue and push legislative action against climate change by speaking in related parliamentary debates.

**Fig. 5:**
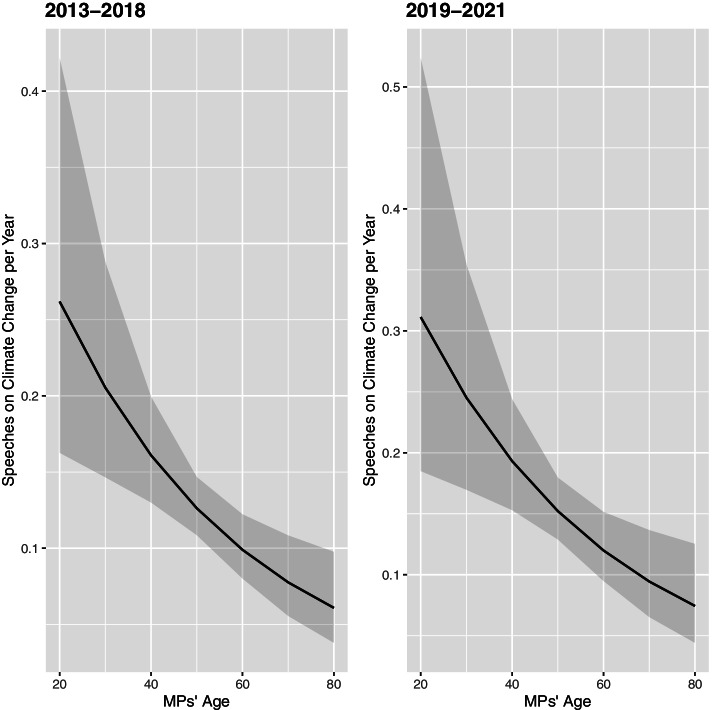
Predicted number of speeches of MPs in parliamentary debates on climate change per year, depending on the age of MPs and differentiated before and after 2019 (the grey shaded area shows the 90% confidence interval)

This finding is confirmed when differentiating between the period between 2013 and 2018 and between 2019 and 2021. While climate change already represented a salient issue for the German population in the five years since 2013, it was only perceived the most pressing issue for a minority. This changed compared to the period between 2018 and 2020, in which a plurality of Germans began to consider topics related to climate change as the most urgent ones (see Fig. 1). The results show that younger MPs give significantly more speeches than older MPs in debates on climate change, which was, however, also the case to a similar same degree in the time period between 2013 and 2018 (see Fig. 5 and Table 2). Contrary to the expectations, there is no evidence that younger MPs — or their parliamentary parties which play an important role in allocating floor time in the German Bundestag (Müller et al. 2021) — are in particular concentrating on climate policy in their legislative work if the public agenda focuses in particular on climate issues. Instead, the results indicate that younger MPs continuously focus more on climate issues than older MPs, possibly to strengthen their own profile and/or that of their party.

**Table 2:** Determinants of the number of MPs’ speeches in debates on climate change, by legislative period

	Model 1 (2013–2018)	Model 2 (2019–2021)
Age	− 0.024^***^ (0.008)	− 0.024^***^ (0.008)
Pop. share below 35 years in electoral district	0.004 (0.024)	0.012 (0.023)
Committee member	2.470^***^ (0.190)	2.086^***^ (0.195)
MP directly elected	0.230 (0.200)	0.457^*^ (0.274)
Female	0.311^**^ (0.158)	− 0.065 (0.182)
Reelected	0.867^***^ (0.174)	0.717^***^ (0.188)
Party seat share	− 0.021^***^ (0.006)	− 0.025^*^ (0.013)
Unemployment rate in electoral district	− 0.064^**^ (0.028)	− 0.028 (0.034)
Constant	− 0.919 (1.060)	− 1.271 (1.047)
Observations	3,143	1,687
Log likelihood	− 1293.996	− 795.392
Theta	0.125^***^ (0.013)	0.241^***^ (0.035)
Akaike Inf. Crit.	2605.992	1608.785

Standard errors in parentheses. Significance levels*:* *p < 0.1, **p < 0.05, ***p < 0.01

## Conclusion

Climate change-related issues have become a salient topic within the German population and therewith the German Bundestag throughout the past decade. This development provides strategic opportunities for office-seeking legislators. The aim of this contribution was to examine whether younger MPs deliver more speeches in parliament on a topic that is of high salience for younger citizens. Against this backdrop, we focused on climate change as an issue that has major implications for the future of younger people and future generations. Arguing within the realm of vote- and career-seeking reasons, younger MPs make use of their age to be perceived as sincere advocates of implementing and promoting policies on climate change. We found that, even when controlling for a number of other factors that are highlighted as decisive by the literature on legislative debates, younger MPs give indeed more speeches in debates on climate change than older MPs. This finding supports our theoretical consideration that younger MPs use their particular personal characteristic to be considered as sincere advocates of policies against climate change and, thus, as representatives of the interests of younger voters and citizens.

We concentrated on individual contributions to parliamentary debates to determine whether younger MPs give more speeches on climate change than older MPs. Needless to say, the focus on parliamentary debates in a single parliament — the German Bundestag — with a dictionary covering not only climate issues, but also environmental issues only offers a restricted perspective. Further research should develop a more specific dictionary that covers all relevant dimensions of climate policy and should adopt a comparative perspective, which is possible given the existing datasets on legislative debates by Rauh and Schwalbach (2020, for a comparative study on how electoral institutions influence emphasizing climate policy by MPs, see Geese and Jordan 2022), and by integrating other options for examining the relationship between MPs and their constituents, e.g., by analyzing social media data like twitter or Facebook entries (e.g., Sältzer 2020). Moreover, the age of an MP depicts a very straightforward and broad indicator for the strategy of an MP to be considered a sincere advocate of climate change policies. More detailed information on the personal background of MPs like his or her family structure, the number of children and grandchildren as well as the MPs’ professional and religious background may offer a more accurate picture on the involvement of an MP in debates and discussions on climate change. However, it is difficult to gather such data, as researchers can only rely on the information MPs provide on their (personal) websites. At the same time, there is obviously no requirement for elected legislators to provide publicly information on, for instance, their family status. While this paper contributes to shed light on the mere involvement, in a next step it would of course be interesting what MPs — in particular the younger ones — actually say in the respective debates on climate change and what position they adopt on climate policy. While there are several computerized methods of content analysis available for measuring the policy positions of the MPs on the basis of their speeches, which would also help for a more precise identification of debates related to climate policy, we leave it to further research to theoretically discuss and to empirically evaluate which factors influence what climate policy position MPs adopt when speaking in parliaments. Finally, one could argue that younger MPs who were already in charge of climate policy-making — for instance as the (junior) minister for environmental affairs in the cabinet — speak less in debates on climate policy since these politicians could be considered as not trustworthy anymore by the voters because of their failure to implement policies that would help to stop climate change. Because of the small number of former cabinet members in charge of climate change that were young, further comparative studies could test this expectation which we briefly outlined here.

The findings presented in this contribution showed that politicians indeed link a characteristic like their age to an issue to address — in this case — younger citizens. In addition to and beyond the personal background of an MP, it is crucial to acknowledge further institutional and contextual features might influence the activity of MPs in climate change debates in parliament. Depending on the parliamentary rules, floor access can be restricted by the parliamentary party leadership or individual MPs can take the floor without the agreement of their parliamentary party leadership. Given that Germany can be considered as a case where the party leadership in parliament is a decisive player when it comes to the question who is allowed to speak (Bäck et al. 2021b; Müller et al. 2021; Proksch and Slapin 2012), the findings presented here could also indicate that the party elite selects younger MPs as speakers in debates on climate change for vote-seeking reasons. That strategy could seek to benefit the party overall and not (only) the respective MP from the possibility to gain more competence on a salient issue if younger MPs speak in debates related to climate change. Since this is an aspect not covered within this paper and with the data at hand, further studies could conduct interviews with younger MPs and members of the parliamentary party leadership to gain more insight on that specific perspective.

## Data Availability

The data used in the manuscript along with all replication material are available in the Harvard Dataverse (10.7910/DVN/FFLNYR).

## Footnotes

1 Very short speeches have shown to have insufficient topical content to be analyzed, such that common practice has developed towards excluding them. Cut-off points vary immensely with some contributions even cutting all speeches below 150 words (Curran et al. 2018). We estimated the empirical models with a cut-off point of 100 words and find that this does not change our results in significant terms.

2 Multiple usages of the same keyword are counted separately.

3 We consider the following words as indicators for debates on environmental issues related to climate change (asterisks indicate that all words with the respective stem are counted as keywords, e.g. “klima*” includes words like “klimawandel” or “klimabewegung”; English translations are provided in parantheses): "erneuerbar*" (“renewable”), "emission*" (“emission”), "klima*" (“climate”), "kohle" (“coal"), "fossil*" (“fossil”), "*erwärmung" (“warming“), "nachhalti*" (“sustainable”), "umwelt*" (“environment”), "*ausstoß" (“emissions”), "grad" (“degree”), "öl" (“oil”), "treibhaus*" (“greenhouse”).

## References

[CR1] Adams J, Somer-Topcu Z (2009). Policy adjustment by parties in response to rival parties’ policy shifts: spatial theory and the dynamics of party competition in twenty-five post-war democracies. Br J Polit Sci.

[CR2] Andeweg RB, Nijzink L, Döring H (1995). Beyond the two-body image: relations between ministers and MPs. Parliaments and majority rule in Western Europe.

[CR3] Bäck H, Debus M (2019). When do women speak? A comparative analysis of the role of gender in legislative debates. Polit Stud.

[CR4] Bäck H, Debus M (2020). Personalized versus partisan representation in the speeches of migrant members of parliament in the German bundestag. Ethn Racial Stud.

[CR5] Bäck H, Debus M, Fernandes JM, Bäck H, Debus M, Fernandes JM (2021). The politics of legislative debates: an introduction. The politics of legislative debates.

[CR6] Bäck H, Debus M, Fernandes JM (2021). The politics of legislative debates.

[CR7] Baumann M (2018). Turning liberal: legislators’ individual preferences and the regulation of preimplantation genetic diagnosis in Switzerland. Swiss Pol Sci Review.

[CR8] Baumann M, Debus M, Müller J (2015). Personal characteristics of MPs and legislative behavior in moral policymaking. Legis Stud Q.

[CR9] Berker LE, Pollex J (2021). Friend or foe? – comparing party reactions to Fridays for future in a party system polarised between AfD and green party. Z Vergl Polit Wiss.

[CR10] Bevan S, Baumgartner FR, Breunig C, Grossman E (2019). Gone fishing: the creation of the comparative agendas project master codebook. Comparative policy agendas: theory, tools, data.

[CR11] Bird K, Saalfeld T, Wüst AM (2010). The political representation of immigrants and minorities: voters, parties and parliaments in liberal democracies.

[CR12] Blumenau J (2021). The effects of female leadership on women’s voice in political debate. Br J Polit Sci.

[CR13] Bol D, Gschwend T, Zittel T, Zittlau S (2021). The importance of personal vote intentions for the responsiveness of legislators: a field experiment. Eur J Polit Res.

[CR14] Bräuninger T, Debus M (2009). Legislative agenda-setting in parliamentary democracies. Eur J Polit Res.

[CR15] Bräuninger T, Debus M, Wüst F (2017). Governments, parliaments and legislative activity. Polit Sci Res Methods.

[CR16] Burden BC (2000). Voter turnout and the national election studies. Polit Anal.

[CR17] Burden BC (2007). Personal roots of representation.

[CR18] Carnes N (2012). Does the numerical underrepresentation of the working class in congress matter?. Legis Stud Q.

[CR19] Carter N, Ladrech R, Little C, Tsagkroni V (2018). Political parties and climate policy: a new approach to measuring parties’ climate policy preferences. Party Polit.

[CR20] Catalano A (2009). Women acting for women? An analysis of gender and debate participation in the British house of commons 2005–2007. Polit Gend.

[CR21] Crawley S, Coffé H, Chapman R (2021) Climate belief and issue salience: comparing two dimensions of public opinion on climate change in the EU. Soc Indic Res:1–19

[CR22] Curran B, Higham K, Ortiz E, Vasques Filho D (2018). Look who’s talking: two-mode networks as representations of a topic model of New Zealand parliamentary speeches. PLoS One.

[CR23] Debus M, Tosun J (2021). The manifestation of the green agenda: a comparative analysis of parliamentary debates. Environ Polit.

[CR24] Eshima S, Smith DM (2021) Just a number? Voter evaluations of age in candidate choice experiments. J Polit. 10.1086/719005

[CR25] Euchner EM, Preidel C (2017). Politicisation without party discipline. A new perspective on Christian democracy in modern times. Parliam Aff.

[CR26] Geese, L, Jordan A (2022) Representative democracy and climate change: do electoral institutions disincentivise politicians’ prioritisation of the issue? Working paper presented presented at the ECPR Joint Sessions 2022 in Edinburgh, Scotland, 19-22 April 2022.

[CR27] Goerres A (2008). The grey vote: determinants of older voters’ party choice in Britain and West Germany. Elect Stud.

[CR28] Grimmer J, Stewart BM (2013). Text as data: the promise and pitfalls of automatic content analysis methods for political texts. Polit Anal.

[CR29] Hájek L (2019). Effects of age and tenure on MPs’ legislative behavior in the Czech Republic. J Legis Stud.

[CR30] Hargrave L, Blumenau J (2021) No longer conforming to stereotypes? Gender, political style, and parliamentary debate in the UK. Br J Polit Sci. 10.1017/S0007123421000648

[CR31] Hobolt SB, De Vries CE (2015). Issue entrepreneurship and multiparty competition. Comp Pol Stud.

[CR32] Höhmann D (2020). When do female MPs represent women’s interests? Electoral systems and the legislative behavior of women. Polit Res Q.

[CR33] Homola J (2021) The effects of women’s descriptive representation on government behavior. Legis Stud Q. 10.1111/lsq.12330

[CR34] Kittilson MC (2011). Women, parties and platforms in post-industrial democracies. Party Polit.

[CR35] Knill C, Tosun J (2020). Public policy: a new introduction.

[CR36] Koch MT, Fulton SA (2011). In the defense of women: gender, office holding, and national security policy in established democracies. J Polit.

[CR37] Laver M, Garry J (2000). Estimating policy positions from political texts. Am J Polit Sci.

[CR38] Meyer TM, Wagner M (2016). Issue engagement in election campaigns the impact of electoral incentives and organizational constraints. Polit Sci Res Methods.

[CR39] Müller J, Stecker C, Blätte A, Bäck H, Debus M, Fernandes JM (2021). Germany: parliamentary debates directed by strong party groups. The politics of legislative debate.

[CR40] Müller WC, Saalfeld T (1997). Members of parliament in Western Europe: roles and behavior.

[CR41] Ohmura T, Bailer S, Meißner P, Selb P (2018). Party animals, career changers and other pathways into parliament. West Eur Polit.

[CR42] Parth AM, Weiss J, Firat R, Eberhardt M (2020). “How dare you!”—the influence of Fridays for future on the political attitudes of young adults. Front Pol Sci.

[CR43] Phillips A (1995). The politics of presence.

[CR44] Proksch SO, Slapin JB (2012). Institutional foundations of legislative speech. Am J Polit Sci.

[CR45] Rauh C, Schwalbach J (2020) The ParlSpeech V2 data set: full-text corpora of 6.3 million parliamentary speeches in the key legislative chambers of nine representative democracies, 10.7910/DVN/L4OAKN.

[CR46] Reher S (2022). Do disabled candidates represent disabled citizens?. Br J Polit Sci.

[CR47] Reynolds A (2013). Representation and rights: the impact of LGBT legislators in comparative perspective. Am Polit Sci Rev.

[CR48] Rovny J, Whitefield S (2019). Issue dimensionality and party competition in turbulent times. Party Polit.

[CR49] Saalfeld T, Bischof D (2013). Minority-ethnic MPs and the substantive representation of minority interests in the house of commons. Parliam Aff.

[CR50] Sältzer M (2020). Finding the bird’s wings: dimensions of factional conflict on twitter. Party Polit.

[CR51] Searing D (1994). Westminster’s world: understanding political roles.

[CR52] Spoon JJ, Hobolt SB, De Vries CE (2014). Going green: explaining issue competition on the environment. Eur J Polit Res.

[CR53] Stockemer D, Sundström A (2018). Age representation in parliaments: can institutions pave the way for the young?. Eur Polit Sci Rev.

[CR54] Stockemer D, Sundström A (2019). Young deputies in the European Parliament: a starkly underrepresented age group. Acta Politica.

[CR55] Strøm K (1997). Rules, reasons and routines: legislative roles in parliamentary democracies. J Legis Stud.

[CR56] Strøm K, Blomgren M, Rozenberg O (2012). Roles as strategies: towards a logic of legislative behavior. Parliamentary roles in modern legislatures.

[CR57] Sundström A, Stockemer D (2021). Conceptualizing, measuring, and explaining youths’ relative absence in legislatures. PS: Pol Sci Polit.

[CR58] Tavits M, Potter JD (2015). The effect of inequality and social identity on party strategies. Am J Polit Sci.

[CR59] Wallis H, Loy LS (2021). What drives pro-environmental activism of young people? A survey study on the Fridays for future movement. J Environ Psychol.

[CR60] Wängnerud L (2009). Women in parliaments: descriptive and substantive representation. Annu Rev Polit Sci.

[CR61] Zittel T, Nyhuis D (2021). The legislative effects of campaign personalization. An analysis on the legislative behavior of successful German constituency candidates. Comp Pol Stud.

